# The structural basis of the talin–KANK1 interaction that coordinates the actin and microtubule cytoskeletons at focal adhesions

**DOI:** 10.1098/rsob.230058

**Published:** 2023-06-21

**Authors:** Xingchen Li, Benjamin Thomas Goult, Christoph Ballestrem, Thomas Zacharchenko

**Affiliations:** ^1^ Wellcome Centre for Cell-Matrix Research, Faculty of Biology, Medicine and Health, University of Manchester, Dover Street, Manchester M13 9PT, UK; ^2^ School of Biosciences, University of Kent, Canterbury CT2 7NJ, UK

**Keywords:** cell adhesion, talin, KANK1

## Abstract

Adhesion between cells and the extracellular matrix is mediated by heterodimeric (*αβ*) integrin receptors that are intracellularly linked to the contractile actomyosin machinery. One of the proteins that control this link is talin, which organizes cytosolic signalling proteins into discrete complexes on β-integrin tails referred to as focal adhesions (FAs). The adapter protein KANK1 binds to talin in the region of FAs known as the adhesion belt. Here, we adapted a non-covalent crystallographic chaperone to resolve the talin–KANK1 complex. This structure revealed that the talin binding KN region of KANK1 contains a novel motif where a β-hairpin stabilizes the α-helical region, explaining both its specific interaction with talin R7 and high affinity. Single point mutants in KANK1 identified from the structure abolished the interaction and enabled us to examine KANK1 enrichment in the adhesion belt. Strikingly, in cells expressing a constitutively active form of vinculin that keeps the FA structure intact even in the presence of myosin inhibitors, KANK1 localizes throughout the entire FA structure even when actomyosin tension is released. We propose a model whereby actomyosin forces on talin eliminate KANK1 from talin binding in the centre of FAs while retaining it at the adhesion periphery.

## Graphical abstract

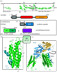

## 1. Introduction

The adhesion of cells to the extracellular matrix (ECM) controls cell migration, proliferation and differentiation [1–3]. The cytoplasmic adapter protein talin controls the ability of cells to adhere to the ECM. Intracellular binding of talin to integrin adhesion receptors activates them and initiates the formation of adhesion complexes, that upon linkage to the force-inducing actomyosin cytoskeleton mature into larger cell–ECM contacts known as focal adhesions (FAs) [2,4]. KANK proteins (isoforms 1–4) are known to bind to talin, but unlike talin, which is ubiquitous throughout the FAs, they localize to a belt region in the periphery of FAs [5,6]. Here they recruit the cortical microtubule stabilizing complex (CMSC) formed of *α* and *β* liprins, LL5*β* and KIF21A, which organizes microtubule plus ends at the cell cortex [6,7]. More detailed mechanistic insight into KANK recruitment and localization requires structural insight; however, the precise structural determinants of this important talin–KANK interaction have been elusive.

**Figure 1.**
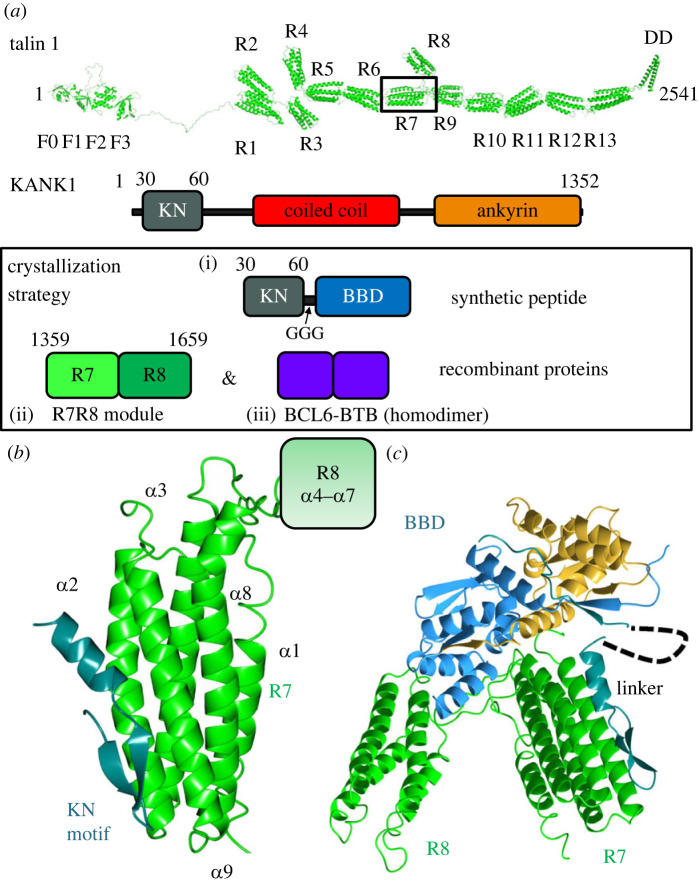
The structure of talin R7 in complex with the KANK1 KN motif. (*a*) (Top) Talin contains an N-terminal FERM domain connected to a rod region (R1–R13) composed of thirteen 4- and 5-helix bundles. The KANK1 binding site is on the R7 helical bundle. KANK1 domain structure (middle) and (bottom) the three protein units used in the crystallization strategy were (i) a KN-BBD (BCL6 binding domain) synthetic peptide, (ii) the R7R8 module, and (iii) the BCL6–BTB homodimer. (*b*) The KN motif (dark green) binds to talin R7 (green) between helices *α*2 and α9 with no change in any of the helical positions. (*c*) The BTB chaperone works by capturing the talin–KANK1 complex in a readily crystallizable lattice. The N-terminal KN motif binds to R7 and the BBD engages the BCL6 lateral groove.

At a structural level, talin contains an atypical N-terminal FERM domain which is linked with a short linker region to the talin rod region (figure 1*a*) [4]. The rod is composed of 13 helical bundles (R1–R13) and a C-terminal dimerization domain (DD) [4]. While the talin FERM domain binds to integrins [8], the helical bundles in the rod bind to actin and a large number of regulatory proteins [9–11]. The association to filamentous actin (F-actin) can be both direct through two actin-binding sites (ABS2, R4-R8 and ABS3, R13-DD) and indirect through the binding and activation of vinculin which also has an ABS [12]. Forces associated with actomyosin activity induce talin conformation changes that can unmask binding sites for vinculin (vinculin binding sites; VBS) and actin (ABS2) [12,13]. We showed previously that constitutively active forms of vinculin that are C-terminally truncated can lock the talin in an activated conformation [13,14]. When expressed in cells, these active vinculin forms, that contain the talin-binding N-terminal Vd1 domain (or equivalent lacking Vd5, vin880), stabilize FAs even when actomyosin-mediated tension is blocked through inhibitors [14].

The talin rod also binds to another class of proteins which bind to the folded rod domains called LD motifs. LD motifs have been identified in multiple talin-binding partners including RIAM [15], DLC1 [16], CDK1 [17] and paxillin [16], and are characteristically small amphipathic α-helices (I/LDxØØxØØ consensus sequence where Ø denotes a hydrophobic residue). These LD motifs commonly pack against talin rod domains using a helix addition mechanism and are best exemplified in multiple structures with the talin R8 4-helix bundle [15–17]. KANK (isoforms 1–4) proteins also contain a predicted LD motif in their KN motif region that binds to the 5-helix bundle R7 (figure 1*a*) [5,6]. However, whereas many of the other binding partners bind to multiple rod domains, KANK binding to talin seems unique since they do not share the promiscuity of binding partners such as paxillin and RIAM. Understanding this novel interaction is therefore important to understand how such specificity for R7 occurs but attempts to crystallize the talin–KANK complexes have been unsuccessful.

In this study, we aimed to gain specific structural detail about the talin–KANK1 interaction and its function in regulating KANK1 localization to FAs. Using a non-covalent crystallization chaperone, we established a technology that allowed us to solve the structure of an engineered complex that contains the authentic R7–KN interface [18]. Surprisingly, we find that the KN motif is not a conventional LD motif, as instead of being a single helix it contains an N-terminal β-hairpin that participates in a novel talin binding interface. Using this new structural information, we designed single-point mutations that disrupt full-length (FL) talin–KANK1 interactions. We show these point mutations completely abolish KANK1 FA localization, demonstrating that the interaction with talin is essential for KANK1 recruitment to cell-matrix adhesion sites. Stabilizing FAs using a constitutively active form of vinculin in parallel with actomyosin inhibitors showed that F-actin directly excludes KANK1 from the core adhesion. Our data lead to a model where actomyosin contractility regulates talin conformation to either promote paxillin–vinculin interactions in the core adhesion or promote KANK1 interactions in the adhesion periphery.

## 2. Results

### 2.1. Determination of the talin–KANK1 complex using a non-covalent crystallization chaperone

KANK proteins share a common overall domain structure, each with an N-terminal KN motif responsible for a direct interaction with the talin rod domain R7 (figure 1*a*). However, despite the biochemical characterization of this interaction, the atomic level detail of this interaction was lacking. Therefore, we set out to determine the crystal structure of the talin–KANK1 complex using synthetic KN peptides mixed with recombinant talin R7R8. Attempts to crystallize the complex using standard screening methods including multiple peptide variants, ligand ratio and protein concentrations failed to produce crystalline material and we postulated that the KN motif peptides directly inhibited crystallographic packing.

To overcome this common bottleneck in protein crystallography we generated a new version of affinity capture crystallography (ACC), a method which uses the homodimeric BTB domain of BCL6 as a non-covalent crystallization chaperone [18]. This and similar approaches use proteins that readily crystallize to donate interfaces and symmetry elements to enable the crystallization of difficult targets [19,20]. The procedure requires the expression of a fusion protein containing the monomeric protein of interest with a C-terminal BCL6 binding domain (BBD) peptide from its natural binding partner nuclear co-repressor 1 (NcoR1). The BBD tag confers a constant high affinity for the BCL6–BTB homodimer that contains two BBD binding sites (lateral grooves; electronic supplementary material, figure S1*a*) which were modified to be primed for crystal contacts in high-ionic-strength conditions. The benefit of this approach is that the chaperone provides an immediate 2-fold symmetry axis that readily packs via multiple potential modes (electronic supplementary material, figure S1*b*). We synthesized a fusion peptide of the mouse KANK1 KN motif (mouse residues 30–60; figure 1*a*; electronic supplementary material, figure S2*a*) linked to the NcoR1 BBD sequence by a short triglycine linker (KN1_BBD_; electronic supplementary material, figure S2*b*). A homogeneous ternary complex of the BCL6–R7R8–KN_BBD_ was made and purified using size exclusion chromatography (electronic supplementary material, figure S2*c*). The resulting complex was readily crystallized (electronic supplementary material, figure S2*d*) and enabled us to collect X-ray diffraction data of the BCL6–R7R8–KN_BBD_ complex to determine the structure by molecular replacement (figure 1*b*).

The crystal structure revealed how the BTB chaperone supported the crystallization of the R7–KN motif complex. Whereas the R7–KN motif interface points toward a solvent channel and is free of contacts (electronic supplementary material, figure S3*a*). The BTB chaperone has donated a back–back interface between BTB homodimers and created a crystallographic tetramer parallel to the *a*-axis, and additional contacts were donated from talin R7 that forms a 3-fold homotrimeric complex on the *c*-axis (electronic supplementary material, figure S3*b*). Overall, the asymmetric unit contained a BCL6 homodimer and a single R7R8 molecule bound to the KN_BBD_ peptide (figure 1*c*). Both the KN motif and the NcoR1_BBD_ regions are well resolved in the *F*_0_-*F*_C_ map, the 2*F*_0_-*F*_C_ map and simulated annealing composite omit maps (electronic supplementary material, figure S4*a*) where they shared a similar B-factor distribution of 136.36 Å^2^ and 139.54 Å^2^, respectively. In the structure, only one of the two lateral grooves of the BTB is occupied due to an unexpected interface between BCL6 and R8 (electronic supplementary material, figure S4*b*) that occludes access to the upper lateral groove sterically restricting corepressor access on a single side. Our strategy defines a new tactic in the determination of challenging protein complex structures and has revealed the structural basis of the talin–KANK1 interaction.

### 2.2. The KN motif contains an N-terminal β-hairpin

**Figure 2.**
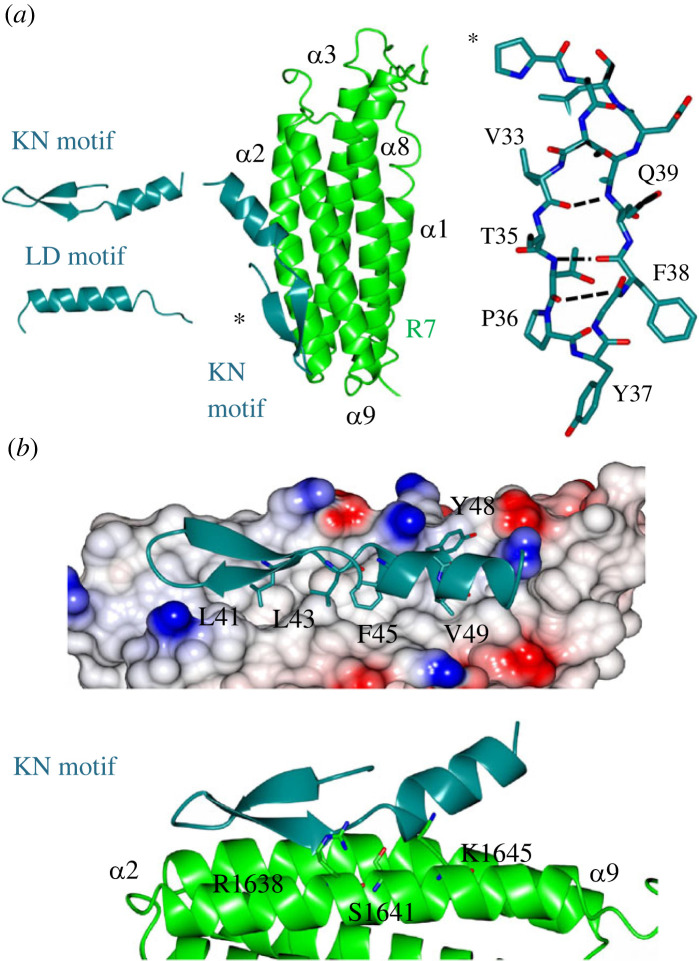
The structure of the talin–KANK1 complex reveals a novel interface. (*a*) (Left) The KN motif is novel and different from other LD motifs. The LD motif of DLC1 is shown for comparison. (Middle) The crystal structure of the talin–KANK1 complex reveals a novel arrangement where intramolecular hydrogen bonds maintain the compact shape of the KN motif. (Right) The KN motif β-hairpin hydrogen bonds. (*b*) The anti-parallel β-strand maintains a rigid hydrophobic interface mediated by L41, L43, F45 and V49 side chains, and with carbonyl side chain bonding donated from talin R1638, S1641 and K1645.

All previously solved complexes with LD motifs have shown the LD motif to adopt only an α-helical conformation (figure 2*a*). By contrast, the KN motif is comprised of a β-hairpin linking to an α-helix. In this arrangement, the anti-parallel β-hairpin is sustained by intramolecular hydrogen bonds between the backbone residues, V33, Q34, T35, P36, F38 and Q39. Whereas most amphipathic helices tend to have only helical propensity in isolation, L41 connects to and stabilizes the C-terminal α-helix by creating a hydrophobic cap with L43 (electronic supplementary material, figure S5*a*), which maintains a talin binding epitope with both charged and hydrophobic faces (electronic supplementary material, figure S5*b*). Interestingly the KN motif structure was also predicted by AlphaFold 2 [21] and structural comparison showed that the predicted structure is topologically identical to the atomic structure (electronic supplementary material, figure S5*c*). The KN motif defines a novel class of talin recognition partners.

### 2.3. The structure of the talin–KANK1 complex reveals a novel way to engage a helical bundle

The currently available structures of LD motifs bound to talin show that the interaction is mediated via the helical portion of the LD motif interacting with the helical bundle. Consistent with the previous model of the talin–KANK1 interaction [6], the KN motif interacts with the hydrophobic *α*2–α9 face of the R7 domain (figure 2). However, the structure reveals two unexpected and novel insights: (1) the KN motif has the opposite polarity being rotated 180̊ and (2) the R7–KN motif interface is driven principally by the KANK1 β-strand that intercalates with the hydrophobic residues on R7. The interaction involves two main regions: firstly the carbonyl backbone of the KN motif β-strand participates in hydrogen bonding with the side chains of S1637, R1638, K1645, T1649 and R1652 on R7 (figure 2*b*), and secondly, the KN motif signature ‘LD’ region, ^41^LDLDF^45^, where the side chains of L41, L43, F45 and V49 occupy complementary hydrophobic cavities that pattern the R7 surface. The side chain of Y48 which was previously modelled as central to the interface with G1404 points away from the binding site in our structure [6] (figure 2*b*); instead this site adjacent to G1404 is occupied by the L43 side chain. Overall, the novelty of the KN motif, and the unique interface it makes with talin R7 explain the reason for its stringent specificity and high affinity, in contrast to simpler α-helical LD motifs such as RIAM and paxillin whose talin rod interactions are multiple. The new structure provided the rationale for the design of structure-based mutations to disrupt the interaction.

### 2.4. KANK1 point mutants that disrupt the talin interaction abolish KANK1 localization to focal adhesions

**Figure 3.**
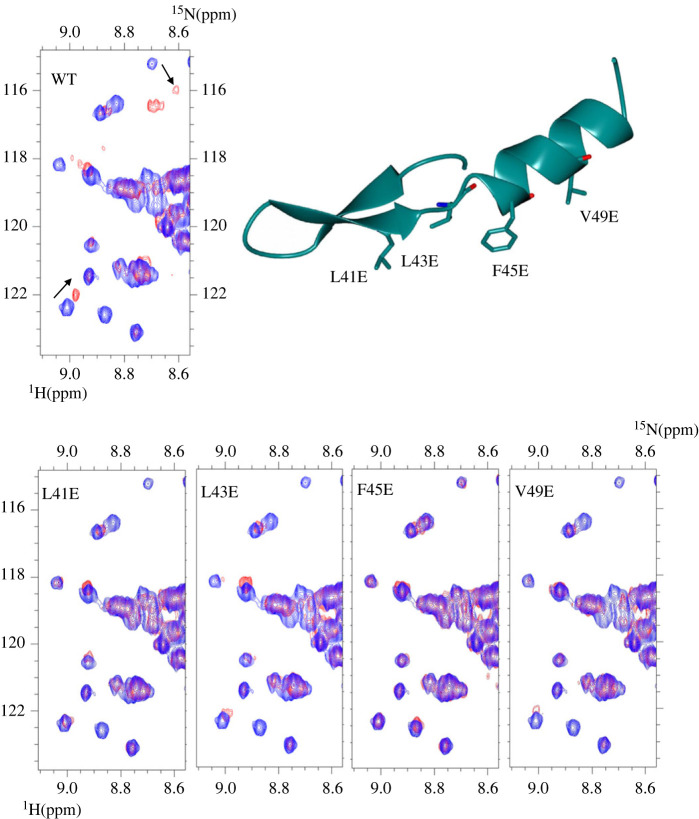
HSQC mapping of the talin–KANK1 interface. ^1^H,^15^N HSQC spectra of 400 µM R7R8 (blue) titrated with a 2:1 molar excess of synthetic KANK1 WT peptide (red) (top). The locations of L41E, L43E, F45E and V49E mutations are shown in the cartoon of the KN motif (right). Bottom: spectra of R7R8 on its own (blue) and in the presence of 2:1 KANK1 peptides (red).

We next explored the effect of charge mutations using nuclear magnetic resonance (NMR). The affinity of the KN motif peptide for R7R8 is tight, *K*_d_ 1.2 µM (electronic supplementary material, figure S5*d*), and heteronuclear single quantum coherence (HSQC) measurements of ^15^N-labelled R7R8 with a 2 : 1 molar excess of KN motif peptide resulted in large chemical shift changes consistent with this high-affinity interaction (figure 3). We next tested variants of the KN motif peptide that were designed to perturb the key contacts identified from the structure. These point mutants introduced single negative charges to replace hydrophobic residues involved in the interface including L41E, L43E, F45E and V49E. While the wild-type (WT) KN motif showed large chemical shift changes, the L41E and F45E mutations produced minimal chemical shift changes demonstrating that the interaction between the KN motif and talin had been abolished (electronic supplementary material, figure S6). Mutations L43E and V49E were also effective but retained partial, albeit attenuated, interactions.

**Figure 4.**
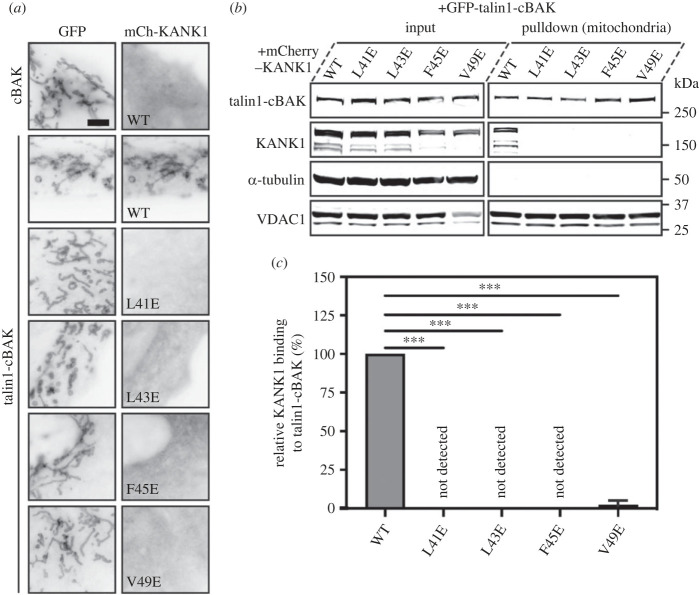
MTS assay and biochemical quantification of talin-KANK1 interactions in cells. (*a*) Co-expression of GFP- and GFP–talin–cBAK with mCherry–KANK1 WT, L41E, L43E, F45E and V49E in NIH3T3 fibroblasts, respectively. The mCherry–KANK1 is recruited to mitochondria as shown by the colocalization with WT GFP–talin–cBAK. Note that all mutations abolish the mitochondrial recruitment of mCherry–KANK1. Scale bar, 5 µm. (*b*) Mitochondria pulldown of KANK1 WT or point mutations in HEK293T cells. Whole-cell lysates (input) and isolated mitochondria were analysed by Western blot with antibodies against GFP, mCherry, α-tubulin and VDAC. (*c*) Quantification of KANK1 mitochondrial pulldown from triplicate experiments. Data are normalized to WT. Error bar is s.d. *** indicates *p* < 0.001 (ordinary one-way ANOVA with Dunnett's multiple comparison test).

To examine the effect of these KANK1 mutations on talin interactions in cells, we next used a mitochondrial targeting system (MTS) which we previously used to screen for defined protein–protein interactions [22]. In this assay, one protein is fused to a small peptide sequence from the pro-apoptotic protein BAK (cBAK) which leads to its insertion into the outer membrane of mitochondria. Proteins that interact with the cBAK-tagged protein will get recruited to mitochondria, and this recruitment can be verified either by visualizing colocalization using fluorescence microscopy (figure 4*a*) or by purification of mitochondria followed by detection of co-precipitates using biochemistry (figure 4*b*,*c*). In such experiments, GFP–talin1–cBAK readily recruits mCherry–KANK1 WT to the mitochondria of both NIH3T3 fibroblasts and HEK293T cells. By contrast, each of the single mutations (L41E, L43E, F45E and V49E) when inserted into KANK1 abolishes GFP–talin–cBAK mediated recruitment (figure 4; electronic supplementary material, figure S7*a,b*). To confirm that the interaction was meditated by the R7 domain, we performed the colocalization assays with truncated talin constructs. While a construct including a rod region starting from R7 to the dimerization motif (GFP–talin1–R7–DD–cBAK) readily colocalized with KANK1, a further truncation comprising R9 to DD (R9–DD) completely abolished colocalization (electronic supplementary material, figure S8*a*). Moreover, the introduction of the R7 G1404L mutation known to prevent KANK1 association with talin (GFP–talin1G1404L–cBAK) also prevented colocalization (electronic supplementary material, figure S8*b*) [6]. These findings demonstrate that the talin–KANK1 interaction is sensitive to disruption by single-point mutations.

### 2.5. KANK1 is enriched in talin-positive areas of the adhesion belt

**Figure 5.**
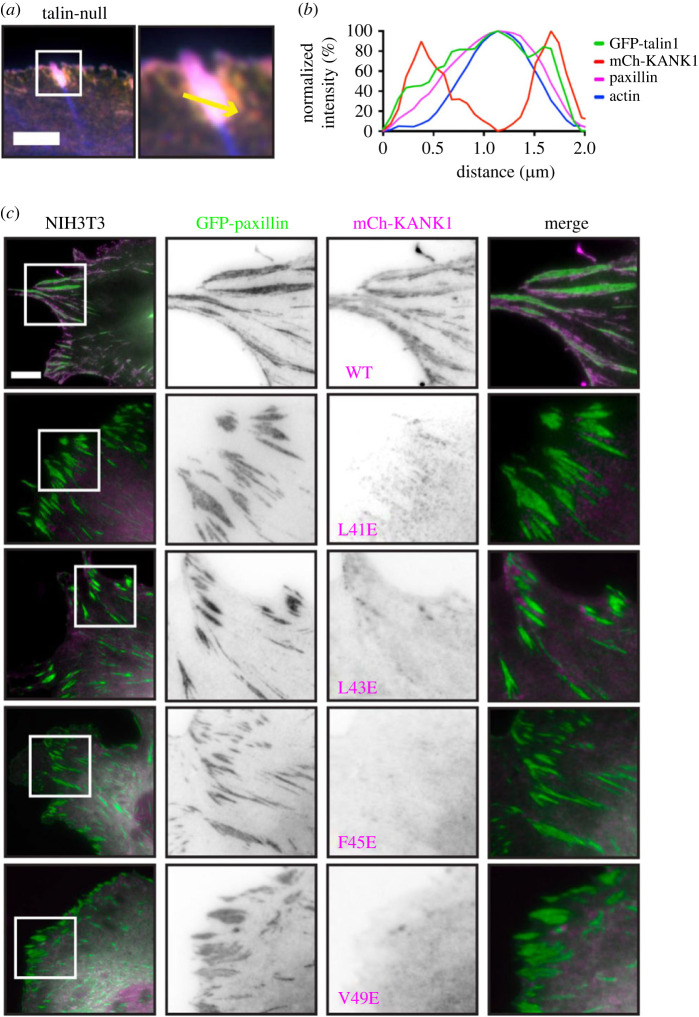
Point mutations in KANK1 abolish adhesion localization. (*a*) Talin null cells were transfected with GFP–talin1 (green), mCherry–KANK1 (red) and immunostained against paxillin (magenta) and actin (phalloidin, blue). Scale bar, 5 µm. (*b*) Line profile (shown by the yellow arrow in *a*) indicates normalized fluorescence intensity levels of proteins from a FA in (*a*). (*c*) NIH3T3 fibroblasts expressing GFP–paxillin and mCherry–KANK1 wild-type and point mutations L41E, L43E, F45E and V49E. The wild-type mCherry–KANK1 is recruited to the FA belt but the mutations abolish this localization. Scale bar, 10 µm.

To examine KANK1 localization to FAs we expressed mCherry–KANK1 together with GFP–paxillin in NIH3T3 cells. While both proteins localize to FAs, central areas of FAs that were strongly positive for paxillin were low in KANK1. Reciprocally, KANK1 localized predominantly to the typical ‘belt-like’ structure surrounding a paxillin-enriched central part of FAs and KANK1-enriched areas at the periphery of FAs were low in paxillin. Next, we visualized GFP–talin1 and mCherry–KANK1 in talin knock-out cells which enabled us to observe talin present in the centre of FAs overlapping with paxillin but also in the periphery overlapping with KANK1 (figure 5*a*; electronic supplementary material, figure S8*c*). Co-staining for F-actin showed that actin stress fibres ended in the core of FAs but not in the peripheral KANK1-positive areas (figure 5*b*). To determine the relative effects of our KANK1 talin-binding mutations on KANK1 localization to FA, we co-expressed WT or mutant mCherry–KANK1 constructs together with GFP–paxillin in NIH3T3 fibroblasts. In these experiments, KANK1 WT readily localized to FAs, and predominantly to the typical ‘belt-like’ structure surrounding the paxillin-enriched central part of FAs. By contrast, all of the KANK1 mutations (L41E, L43E, F45E and V49E) abolished localization to FAs and the FA belt (figure 5*c*). Residual mutant KANK1 proteins still localized to regions outside of FAs that were negative for talin but this was likely due to dimerization with endogenous KANK proteins. The efficiency of our point mutations in abolishing KANK1 localization to adhesion structures demonstrates that KANK1 localization to FAs solely depends on its interaction with talin. Moreover, the overlapping distribution of KANK1 with talin in the adhesion belt but the absence of KANK1 from the FA centre that connects with actomyosin suggested the possibility of two populations of talin. One that links to actin, which abolishes KANK1 binding, and a second one which localizes to the FA periphery that is devoid of actin.

### 2.6. Dissecting mechanosensitive contributions to KANK1 localization

**Figure 6.**
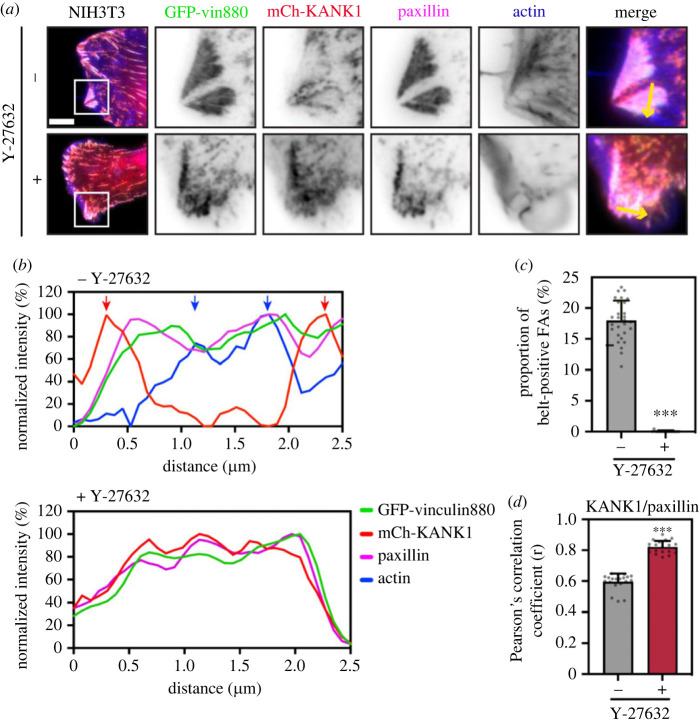
Actomyosin controls the localization of talin–KANK1. (*a*) NIH3T3 fibroblasts expressing GFP–vin880, mCherry–KANK1, and immunostained against paxillin and actin. Cells were treated for 60 min with Y27632 (+) or water (−) with associated line profiles (shown by yellow arrows) shown in (*b*). Distinct peaks of KANK1 and actin are indicated by red or blue arrows. Scale bar, 10 µm. (*c*) Percentage of belt-positive FAs in Y27632 (+) or water (−) treated cells. FAs with a size over 0.3 µm^2^ were counted. −: 17.90 ± 3.27% (*n* = 28); +: 0.02 ± 0.10% (*n* = 17). (*d*) Pearson's mean correlation coefficient of KANK1/paxillin overlap in Y27632 (+) or water (−) treated cells. 20 individual images (40 × 40 µm^2^) of FA area from each group were measured. −: *r* = 0.59 ± 0.05; +: *r* = 0.82 ± 0.04. Error bars are s.d. *** indicates *p* < 0.001 (Welch's *t*-test).

Previous reports have shown that forces on talin unmask VBS in the talin rod [13]. We, therefore, hypothesized that actomyosin-induced tension might unfold R7 to expose the VBS in R7 and binding to vinculin would compete with KANK1 at the central parts of FAs. However, experiments in vinculin null MEFs showed similar proportions of KANK1 localizing to adhesion belts as in control cells (electronic supplementary material, figure S9*a*). As vinculin does not affect KANK1 localization, we next examined whether actomyosin forces themselves have a direct impact in triggering the shift of KANK1 from the actin-rich FA centre to the periphery. To explore this possibility we expressed a tailless, constitutively active vinculin construct, vin880, together with KANK1 in NIH3T3 fibroblasts and co-stained these cells for paxillin and actin. Vin880 has been previously shown to produce dramatically enlarged adhesions by maintaining the talin–integrin complex in an activated state which maintains stable FAs even in the presence of inhibitors that block actomyosin-mediated tension [14]. As shown in figure 6*a*, in the control group vin880 produced enlarged adhesions with about 20% adhesions linked to stress fibres showing KANK1 localization to the FA belt. By contrast, in cells expressing vin880 treated with ROCK inhibitor Y-27632, KANK1 colocalized with paxillin in FAs throughout the whole FA structure (figure 6*a*,*b*). Measurement of paxillin/KANK1 colocalization using Pearson's correlation coefficient showed this difference between the control and Y-27632 treated group was significant (figure 6*c*,*d*; electronic supplementary material, figure S10). These data demonstrate that actomyosin-mediated tension modulates KANK1 localization to FAs with increased actomyosin contractility preventing KANK1 localization to the central part of adhesions.

### 2.7. The talin–KANK1 connection organizes the assembly of the CMSC

**Figure 7.**
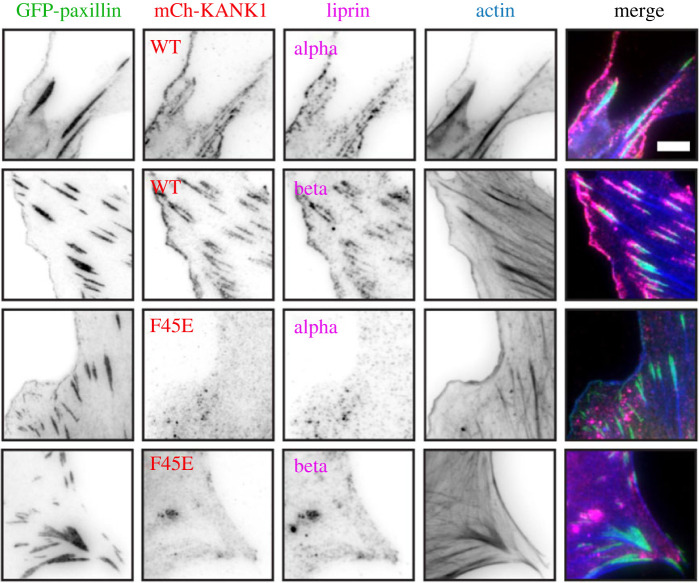
The talin–KANK1 connection controls CMSC assembly. NIH3T3 fibroblasts were co-transfected with GFP–paxillin and either mCherry–KANK1 WT or F45E, and immunostained against either α- or β-liprin (magenta) or F-actin (phalloidin, blue). Scale bar, 5 µm.

As KANK proteins are central components of the CMSC, we next sought to determine the effect of our F45E KANK1 mutation on the localization of *α*/*β* liprin proteins. In line with previous findings our analysis of NIH3T3 cells showed that both *α*/*β* liprins and KANK1 decorate both the cellular cortex and the adhesion belt [6,7] (figure 7). Introduction of KANK1 F45E mutation showed loss of *α*/*β* liprin from both the adhesion belt and the cellular cortex. These findings demonstrate that the talin–KANK1 connection is vital for the ordered assembly of the CMSC both around and recruited to adhesions.

## 3. Discussion

The interaction of talin–KANK1 is critically involved in the recruitment of microtubules to FAs [5,6]. Yet structural details of this important complex have been elusive. In this study, we developed a novel non-covalent chaperone approach to determine the structure of the talin–KANK1 complex. This structure revealed that the KN motif contains a β-hairpin connected to an α-helix that binds the talin R7 domain with high specificity. Our data enabled us to design single-point mutations that abolish the interaction. We confirmed that KANK1 binding to talin is essential for its localization to FAs but also demonstrated that talin engagement with actomyosin prevents KANK1 binding to talin.

Determining the structure of the talin–KANK1 complex relied on the development of a new crystallographic approach that allowed us to resolve authentic binary protein–protein complexes. The use of this method enabled us to map the R7–KN motif interface and crucially revealed that the KN motif represents a compact novel talin binding partner comprising a β-hairpin that stabilizes an α-helical region. There are nine 5-helix bundles in the talin rod of which only R7 and R11 have so far been shown to bind LD motifs. RIAM binds R11 through a conserved charge–charge interaction facilitating the helical packing of an LD motif against the helical bundle similar to how it engages the 4-helix bundles [16]. R7 does not bind KANK1 in this way; instead, the β-hairpin of the KN motif engages a hydrophobic groove localized between *α*2 and α9 on the R7 surface, with a three-dimensional epitope containing the signature ^41^LDLDF^45^ sequence. This β-hairpin forms a helical cap, stabilizing the helical conformation of the C-terminal region and enhancing its affinity and specificity for R7. Interestingly, this sequence also forms part of the predicted KANK nuclear export sequence (NES), the evolutionary progenitors of the LD motif from whom they diverged 800 million years ago [23,24]. It will be interesting to understand in future studies how the KN motif and LD motif have diverged in evolutionary terms to engage talin by independent binding modes.

Fluorescence polarization measurements confirmed previous observations that the KN motif is the highest affinity talin rod binder identified to date. The crystal structure enabled precise point mutants to be designed that disrupt the interaction that we validated using 2D-NMR *in vitro* and a mitochondrial targeting assay in cells. The fact that these mutations prevent KANK1 localization to FAs demonstrated that the KANK1 interaction with talin is essential for its recruitment to cell adhesion sites. This observation is in line with previous reports showing that mutations in talin R7 similarly abolish KANK recruitment to FAs [6], but rules out the hypothesis that the disordered, coiled coils or ankyrin repeat regions may help retain it in adhesion structures [5,6].

In previous studies, the differential distribution of talin and KANK proteins in adhesion sites left speculation about the contribution of other proteins in KANK binding at adhesions. However, a more detailed analysis of the fine distribution of talin and KANK1 showed that talin is also present in the, sometimes rather striking, belt area that KANK1 occupies around FAs. Our results show that the area of FAs highest enriched for F-actin is lowest for KANK1 and vice versa. Previous studies already forwarded the hypothesis that actomyosin negatively impacts KANK localization to adhesions [5,6]. One study found that *in vitro* F-actin and KANK2 compete for the ABS2 region explaining reduced force transmission integrin-mediated adhesions [5], and another observed that KANK gradually occupied the remaining adhesions upon actomyosin inhibition [6]. This led us to explore the relation between the mechanical state of talin, KANK1 localization and the relative contribution of vinculin and F-actin.

Vin880, a constitutively active vinculin construct, maintains FA structures even in the presence of ROCK1 inhibitors but abolishes the conformational changes of actomyosin-induced tension. Our data demonstrated that in the absence of F-actin KANK1 will colocalize perfectly with paxillin and vin880. Mechanistically, and in line with our finding in vinculin null cells, our data rule out the mechanical recruitment of vinculin as a driver of KANK1 adhesion exclusion and highlight the function of F-actin. They also demonstrate that the R7R8 double domain module in talin is folded in these experiments and able to participate in protein interactions that would be thought to be mutually exclusive with vinculin binding. Previous reports have shown that increasing the hydrophobic core of domains, such as R3 [4], can stabilize the fold. Therefore it may be possible that the hydrophobic interface of the R7–KN motif increases the mechanical stability of R7 to permit recruitment and inhibit the talin–vinculin association [25].

KANK1 proteins are part of the CMSC that decorate the leading edge and cellular cortex as well as the adhesion belt [6,7]. Therefore, we sought to examine the localization of *α*/*β* liprin proteins in response to our F45E mutation. Our data demonstrated that this mutation abolished both adhesion localization and also strikingly *α*/*β* liprin organization at the lamellipodia. Our data highlight the reciprocal cross-talk between FA and the CMSC and how talin maintains both assemblies. Overall, our findings have advanced crystallography and provided atomic-level detail about the elusive talin–KANK interaction. In future, this insight will facilitate the design of small molecular inhibitors to disrupt the talin–KANK1 axis, which given the importance of KANK proteins in disease, will enable precise dissection of this important linkage.

## 4. Material and methods

### 4.1. Protein expression and purification

Mouse talin-1 (P26039) R7R8 was expressed and purified as described previously [9]. The BCL6 chaperone was expressed and purified as described previously [18]. Constructs were verified independently by sequencing.

### 4.2. Synthetic peptides

Peptides were purchased from GLBiochem (Shanghai). Peptides include the KN1_BBD_ fusion ^1^PYFVETPYGFQLDLDFVKYVDDIQKGNTIKKGGGGITTIKEMGRSIHEIPR^51^ and the KANK1 KN motif (UniProt E9Q238) ^30^PYFVETPYGFQLDLDFVKYVDDIQKGNTIKKC^60^ (for FP), and^30^PYFVETPYGFQLDLDFVKYVDDIQKGNTIKK^60^ (for NMR). The following peptides were used for NMR screening: L41E ^30^PYFVETP YGFQEDLDFVKYVDDIQKGNTIKK^60^, L43E ^30^PYFVETPYGFQLDEDFVKYVDDIQKGNTIKK^60^, F45E ^30^PYFVETPYGFQLDLDEVKYVDDIQKGNTIKK^60^ and V49E ^30^PYFVETPYGFQLDLDFVKYEDDIQKGNTIKKC^60^.

### 4.3. Fluorescence polarization assay

For determination of the WT-KANK1 binding constant the BODIPY-TMR coupled peptides dissolved in PBS (137 mM NaCl, 27 mM KCl, 100 mM Na_2_HPO_4_, 18 mM KH_2_PO_4_, pH 7.4), 5 mM TCEP and 0.05% (v/v) Triton X-100 were used at a final concentration of 0.5 µM. Uncoupled dye was removed using a PD-10 gel filtration column (GE Healthcare). Fluorescence polarization measurements were recorded on a BMGLabTech CLARIOstar plate reader and analysed using GraphPad Prism (v. 6.07). *K*_d_ values were calculated by nonlinear curve fitting using a one-site total and nonspecific binding model.

### 4.4. Nuclear magnetic resonance

NMR spectra were collected on a Bruker Avance III 800 MHz spectrometer equipped with CryoProbe. Experiments were performed at 298 K in 20 mM sodium phosphate (pH 6.5) and 50 mM NaCl, 3 mM β-mercaptoethanol with 5% (v/v) ^2^H_2_O.

### 4.5. X-ray crystallography

Initial sparse matrix crystal approaches using the strategies described previously for R7R8 complexes failed to produce any crystalline material, as did further modulation of protein/peptide concentration. We used a synthetic peptide containing the NCoR1_BBD_ connected via a triglycine linker to the C-terminus of the KANK1 KN motif (residues 30–60) peptide ^1^PYFVETPYGFQLDLDFVKYVDDIQKGNTIKK-GGG-GITTIKEMGRSIHEIPR^51^. This KN1_BBD_ peptide facilitated the formation of a ternary R7R8–BCL6–KN_BBD_ complex with the BCL6 non-covalent chaperone that was readily purified by size-exclusion chromatography. The complex was concentrated to 10 mg ml^−1^ and used for crystallographic screening in 20 mM Tris (pH 7.4), 150 mM NaCl, and 3 mM β-mercaptoethanol. Crystals were obtained by conventional sparse matrix screening sitting drop vapour diffusion with plates dispensed using a Mosquito Liquid Handling robot (SPT Labtech) with a 1 : 1 precipitant-to-precipitate ratio in 400 nl drops. Crystals were obtained in 1 M ammonium sulfate, 0.1 M CHES (pH 9.5), 0.2 M NaCl, 6% glycerol and typically after approximately 3 weeks and before data collection vitrified in mother liquor containing 20% glycerol. Diffraction data were collected on I03 Diamond Light Source using the automated collection mode and integrated using XDS/SCALA; resolution cut-off was determined by CC_1/2_ at 3.4 Å (0.289) [26,27]. Crystals adopted space group H32 and the structure of the complex was solved by molecular replacement using PHASER [28] with the template structure of the BCL6–NCoR1_BBD_ complex (PDB:6XYX). After molecular replacement electron density of both R7, R8 and the KN motif were visible allowing the placing of R7 and R8 using PHASER. The KN motif, given the resolution range, was impressively well resolved (electronic supplementary material, figure S4*a*). To model the KN motif we traced the polyalanine backbone using the routine modelling tools in COOT [29]. Following rounds of iterative refinement using PHENIX [30], we unambiguously assigned the side chain moieties. Data reduction statistics and refinement information are shown in table 1 and coordinates and structure factors were deposited to the PDB with the accession code 8AS9. Diffraction images have been made publicly available on Zenodo https://doi.org/10.5281/zenodo.7863237 [31].

**Table 1.** 

beamline	I03 Diamond
detector	DECTRIS EIGER2 XE 16M
wavelength (Å)	0.97625
resolution range (Å)	61.89–3.40 (3.58–3.4)
space group	R32:H
*a, b, c* (Å)	207.02, 207.02, 151.86
*α, β, γ* (°)	90, 90, 120
unique reflections	17 332 (2505)
completeness (%)	100 (100)
multiplicity	20.9 (21.8)
mean (I) CC_1/2_	0.992 (0.289)
<*I*/*σ*(*I*)>	5.1 (0.5)
*R*_merge_ (*I*)	0.641 (6.191)
*R*_p.i.m_ (*I*)	0.143 (1.356)
refinement	
RMSD bonds (Å)	0.003
RMSD angles (^o^)	0.880
*R*_work(%)_	24.50 (39.32)
*R*_free(%)_	28.60 (40.27)
no. of atoms	
total	4704
macromolecule	4678
solvent	26
Ramachandran	
favoured (%)	94.43
allowed (%)	4.73

*R*_free_ was calculated using 5% of data isolated from the refinement for cross-validation. The highest-resolution shells are shown in parentheses. TLS parameters used chains A, B, C and D.

### 4.6. Cell lines and transfections

NIH3T3 mouse fibroblasts and HEK293T human epithelial cells were obtained from the American Type Culture Collection (ATCC). The vinculin null and WT mouse embryonic fibroblasts (MEFs) originate from the Eileen Adamson laboratory [32]. All cells were maintained in Dulbecco's Modified Eagle Medium (DMEM; Sigma) supplemented with 10% fetal bovine serum (FBS, Gibco), 1% L-glutamine (Sigma) and 1% non-essential amino acids (Sigma). Talin1&2 null cells [12] were cultured in DMEM F-12 (Gibco) supplemented with 10% FBS, 1% L-glutamine, 1% non-essential amino acids and 15 µM HEPES (Sigma). All cells were cultured at 37̊C supplied with 5% CO_2_ and 95% humidity. Transient transfections were performed using Lipofectamine LTX with Plus Reagent (Invitrogen) to NIH3T3 cells, Lipofectamine 2000 (Invitrogen) to talin null cells, and jetPRIME reagent (Polyplus) to MEFs and HEK293T cells, as per the manufacturer's instructions.

### 4.7. Plasmids preparation and site-directed mutagenesis

For the construction of mCherry-KANK1, human FL-KANK1 (generous donation from the Bershadsky laboratory) cDNA was tagged in the C-terminal site with pmCherry (Clontech) by restriction digestion. Point mutations (L41E, L43E, F45E and V49E) were introduced in mCherry-KANK1 by site-directed mutagenesis (NEB) using the following oligonucleotides: TGGTTATCAAgaAGACTTAGATTTCCTCAAATATG and TAGGGGGTCTCCACAAAG for L41E; TCAACTAGACgaAGATTTCCTCAAATATGTG and TAACCATAGGGGGTCTCC for L43E; AGACTTAGATgaaCTCAAATATGTGGATG and AGTTGATAACCATAGGGG for F45E; CTCAAATATGaGGATGACATACAG and GAAATCTAAGTCTAGTTGATAAC for V49E. Generation of GFP–talin1–cBAK and GFP–cBAK was as previously described [22]. G1404L was introduced on GFP–talin1–cBAK by the method above using CAAGGTCCTActtGAGGCCATGACTGG and GAGTTCTCCATGACACTG. To generate GFP–talin1 R7DD–cBAK and GFP–talin1 R9DD-cBAK constructs, R7-DD (3555 bp) and R9-DD (2661 bp) were amplified from talin-1 (*Mus musculus*). Restriction digestion with XhoI and HindIII FastDigest enzymes (Thermo Scientific) was used to linearize GFP–cBAK. DNA assembly was performed to join R7-DD and R9-DD to linearized the GFP–cBAK vector using the NEBuilder HiFi DNA assembly kit (NEB).

### 4.8. Antibodies and reagents

For fixed cell imaging, cells were cultured in glass-bottom dishes (IBL) coated with bovine fibronectin (Sigma) at a final concentration of 10 µg ml^−1^. Samples were fixed in 4% paraformaldehyde (PFA, Sigma), and warmed to 37̊C, for 15 min before being washed three times with PBS. For immunofluorescence staining, samples were permeabilized at room temperature with 0.5% Triton X-100 (Sigma) for 5 min before being washed three times. The primary antibody rabbit anti-paxillin (clone Y113, ab32084, Abcam) was used at a dilution of 1:200 (in 1% BSA), rabbit anti-liprin α (14175-1-AP, Proteintech) was used at 1:200, and rabbit anti-liprin β (11492-1-AP, Proteintech) was used at 1:200. Secondary antibody Alexa Fluor Plus 647 goat anti-rabbit (Invitrogen) was used at a dilution of 1:500. Actin was visualized using Alexa Fluor Plus 405 Phalloidin (1:500, Invitrogen). Y-27632 (Tocris Bioscience) was diluted in dH_2_O and used at a final concentration of 50 µM. Before use, the stock was diluted in a pre-warmed medium before being added to cells. Mitochondria isolation from HEK293T cells was performed after 24 h of cell transfection using the Qproteome Mitochondria Isolation Kit (QIAGEN). Cell lysis and mitochondria homogenization were conducted as per the manufacturer's instructions. The purified mitochondrial were stored at −80̊C.

### 4.9. Microscopy

Images of fixed samples in PBS were acquired at room temperature using an Olympus IX83 inverted microscope equipped with a 60×/1.42 PlanApo N oil objective and a QImaging Retiga R6 CCD camera, controlled by Metamorph software. Samples were illuminated using LEDs (UV/Cyan/Green-Yellow/Red, Lumencor) for fluorescence excitation; a Sedat filter set (DAPI/FITC/TRITC/Cy5, Chroma, 89000) was used.

### 4.10. Protein extraction and Western blot

Protein samples were extracted from cells and mitochondria, respectively, using RIPA lysis buffer (Chromotek) supplemented with protease inhibitors. Protein samples were diluted in LDS sample buffer (4×, Invitrogen) supplemented with sample reducing agent (10×, Invitrogen). Samples were heated at 95̊C for 5 min before loading on a 4–12% gradient Bis-Tris gel (Invitrogen). MOPS SDS running buffer (Invitrogen) was used and supplied with antioxidants (Invitrogen). The gel was soaked in running buffer and run at 160 V for 75 min. The gel was transferred to a 0.45 µm nitrocellulose membrane (Cytiva) and protein at 30 V for 150 min, 4̊C. The membrane was blocked for 1 h in 5% skimmed milk (Sigma) in PBS-Tween 20 (0.1%, Sigma). The membrane was probed for anti-GFP (ab183734, abcam), anti-mCherry (1C51, ab125096, abcam), anti-VDAC1 (ab15895, abcam) and anti-α tubulin (DM1*α*, T6199, Sigma), diluted 1 : 10 000, 1 : 3000, 1 : 1500, 1 : 1500, respectively, in 5% milk (PBS-Tween). Primary antibody signal was detected using goat anti-mouse IgG conjugated to IRDye 680RD (ab216776, abcam) and goat anti-rabbit IgG conjugated to IRDye 800CW (ab216773, abcam) secondary antibodies, diluted 1 : 15 000, imaged with an Odyssey CLx imaging system (LI-CO Biosciences).

### 4.11. Analysis of cell adhesions

FIJI-ImageJ [33] software was used to process all images. Cell-matrix adhesion size was quantified as described previously [12], by subtracting background signal using a rolling ball algorithm, followed by thresholding to select adhesion structures and the Analyze Particles function to quantify adhesions. The line intensity profile of adhesion was generated using the Plot Profile function. The intensity profile was then normalized between 0 and 100% by dividing the plot value by the maximum value and then multiplying by 100. Pearson's correlation coefficient of fluorescence signals (40 × 40 µm^2^ square adhesion area) was measured by subtracting the background signal, followed by automatic thresholding and colocalization analysis using the Bioimaging and Optics Platform (BIOP) version JACoP plugin [34].

### 4.12. Graphs and statistical analysis

All graphs and statistical analyses were carried out using Prism 9 (GraphPad). Where appropriate, statistical significance between two individual groups was tested using an unpaired *t*-test with Welch's correction. An ordinary one-way analysis of variance (ANOVA) followed by Turkey's multiple comparison tests was performed to test for significance between tests or more groups. Data distribution was tested for normality using a D'Agostino & Pearson omnibus normality test; a *p* value >0.05 was used to determine normality. Data are presented as mean ± standard deviation (s.d.). A *p* value of 0.05 or below was considered statistically significant. * *p* < 0.05, ** *p* < 0.01 and *** *p* < 0.001.

## Data Availability

Atomic coordinates and structure factors have been deposited to the PDB with the accession code 8AS9. The data are provided in the electronic supplementary material [35].
